# Metabolomics of Ocular Tissues with High and Low Metabolic Activity

**DOI:** 10.3390/metabo16030167

**Published:** 2026-03-01

**Authors:** Jack V. Greiner, Thomas Glonek

**Affiliations:** 1 The Schepens Eye Research Institute of Massachusetts Eye & Ear Infirmary, 20 Staniford St., Boston, MA 02114, USA; 2 Department of Ophthalmology, Harvard Medical School, Boston, MA 02114, USA; 3 Clinical Eye Research of Boston, Boston, MA 02114, USA; tglonek@rcn.com

**Keywords:** adenosine triphosphate (ATP), crystalline lens, metabolomics, nucleoside triphosphates (NTP), ^31^P NMR, ^31^P spectral modulus, retina

## Abstract

**Highlights:**

1. This report uses ^31^P nuclear magnetic resonance spectroscopy to study metabolomics focusing on adenosine triphosphate (ATP), nucleoside triphosphates (NTP), and the ^31^P spectral modulus in the crystalline lens and sensory retina.
2. An unexplainably high millimolar concentration of adenosine triphosphate (ATP) exists in the crystalline lens which is similar to that found in the retina. This observation is perplexing because the lens is one of the most metabolically quiescent tissues in the body, whereas the retina is one of the most metabolically active tissues in the body.
3. This study compares phosphorus metabolomics and related metabolic indices in the crystalline lens and retina measuring their metabolic health status.
4. The ^31^P spectral modulus, a measure of overall metabolic health status, is similar in both the lens and the retina.
5. These are novel findings addressing the metabolomics of phosphate metabolism associated with metabolically diverse eye tissues.
6. The high mM nucleoside triphosphate concentration in the lens and retina is hypothesized to function as a hydrotrope, preventing the protein aggregation that results in age-related cataractogenesis and macular degeneration.

**Abstract:**

Background/Objectives: An unexplainably high millimolar (~3 mM) concentration of adenosine triphosphate (ATP), herein designated as nucleoside triphosphate (NTP), exists in the crystalline lens even though all of the known functions of NTP combined require only micromolar (μM) concentrations. Since the lens is one of the most metabolically quiescent tissues in the body and the retina is one of the most metabolically active tissues in the body, we compared their phosphorus metabolomics and related metabolic indices that measure their metabolic health status. As such, the purpose of this report was to compare the NTP concentrations in lenticular and retinal tissues and the metabolic indices that include NTP as well as their phosphorus-31 spectral modulus (PSM). Methods: Known phosphatic metabolic profiles of rat lenses and retinas were compared and quantified in mole % phosphorus using phosphorus-31 nuclear magnetic resonance spectroscopy. Metabolic indices measuring health status, where ATP is a principal component, were calculated, including the PSM. Results: In this secondary analysis, the NTP concentration calculated in the lens was 41.0% of the total phosphate detected, whereas it was similarly 37.6% in the sensory retina. The PSM values were 1.28 for the lens and similarly 1.42 for the retina. Conclusions: Due to the lens tissue’s low quiescent metabolic activity, one might expect the NTP concentration to be lower in the lens than in the highly metabolically active retina: a similar difference is expected in the PSM. However, this was not the case with the mM concentrations of NTP in both the lens (≥2.3 mM) and the retina (2.4 mM). The similarly high mM NTP concentration coupled with the PSM-calculated measure of metabolic health in these tissues is a novel finding. The novel findings of such similarly high concentrations of NTP in these metabolically diverse eye tissues further support and are consistent with the hypothesized role of NTP as a hydrotrope, preventing protein aggregation resulting in age-related cataractogenesis and age-related macular degeneration.

## 1. Introduction

The accepted intracellular role of nucleoside triphosphates (NTP), principally adenosine triphosphate (ATP) and, in the retina, also guanosine triphosphate (GTP), performs along with ATP as the energy currency of cells and tissues [1]. [Henceforth, we will refer to all nucleoside triphosphate signals as NTP because, with the present phosphorus-31 nuclear magnetic resonance (^31^P NMR) technology and magnetic field strength, we are typically unable to spectroscopically resolve GTP, cytosine triphosphate (CTP), and uridine triphosphate (UTP) from the ATP spectral signal.] An exception to this is the GTP, CTP, and UTP signals in the retina [2]. The presence of a high 2.4 millimolar (mM) NTP concentration in the highly metabolic retinal tissue [2] and the metabolically quiescent crystalline lens tissue/organ [3], also maintaining a high > 2.3 mM NTP concentration, supports the hypothesis of another major role of intracellular NTP [4].

Considering the known roles of NTP (see below), the NTP concentration measurements we first reported in the metabolically quiescent intact lens organ were three orders-of-magnitude greater than those required for metabolic activities [3,5]. Moreover, further support confirming this possibility of an alternative function for high concentrations of NTP was suggested by our discovery of the influence of water surrounding the ATP tripolyphosphate side chain [6]. This chance discovery was made using ^31^P NMR in the detection of changes in spectral line widths while making measurements on the intact living lens organ [6,7]. With these findings, we hypothesized that the high-millimolar NTP concentration was being produced for another purpose [4]. These discoveries become especially important given that the production of intracellular high-energy phosphates in living tissues and organs in biology and nature is known to be conservative. As such, excess quantities of NTP are unlikely to be produced and simply stored.

### Cellular NTP Functions [8]

- A molecular carrier of intracellular energy;
- The ultimate metabolic source of high-energy phosphate bonds;
- The parent moiety giving rise to vitamin dinucleotides and other cofactors^a^;
- A coenzyme;
- An allosteric enzyme regulator for modulating protein activities;
- A substrate for the first stage of protein synthesis;
- A modifier of the intracellular milieu;
- The principal metabolite for cellular energy transduction mechanisms;
- The transport of macromolecules, such as proteins, into and out of cells;
- A phosphorylating agent in the phosphate regulation of transmembrane proteins;
- The provider of adenine, one of the four letters of the genetic code;
- A molecule that participates in the signaling of key bioprocesses;
- A transmitter in intercellular purinergic signaling.

[^a^NADH, FAD, CoA, nucleoside diphospho-sugars, etc.]

Patel et al. [9] reported similarly high mM quantities of ATP measured in tissue homogenates. This finding in tissue homogenates, coupled with our earlier report of a similarly high mM concentration of ATP in an intact living organ [3], reinforced our belief that the high mM NTP was being produced for another purpose. Multiple experiments using extracts of tissue homogenates demonstrated that at high concentrations, NTP functions as a hydrotrope [9]. Hydrotropes can solubilize proteins [10] and prevent protein aggregation. Taken together, the aforementioned findings supported the hypothesis presented in the intact living lens organ [4], extolling the likelihood that the high concentration of NTP (~3 mM) in the lens is required in order to prevent cataractogenic protein aggregation [11]. Protein aggregation results in optical dysfunction with consequent loss of functional lens transparency. Prevention of protein aggregation has been shown not only to maintain tissue function and physiological homeostasis in the lens [12,13], but also to mitigate dysfunction and cell death in multiple degenerative diseases, such as retinal age-related macular degeneration [11,14,15,16,17,18].

We present comparisons of both phosphatic metabolites and, most specifically, NTP between tissues with high and low metabolic activity, as well as computed NTP-related ratios representing metabolic health status. The high concentration and involvement of NTP, the tripolyphosphate spectral bands of the nucleoside triphosphates, of which ATP is the greatest component in the ^31^P NMR spectroscopic profile of all tissues, would be expected to be of influence. Although in our previous reports we have presented numerous phosphatic metabolic ratios [19], herein, we have restricted computations to those metabolic ratios including NTP and one other phosphatic metabolite (NTP/NDP, NTP/Pi, and PCr/NTP) and those ratios with more than one phosphatic metabolite that include the energy charge of the adenylate system (AEC) [20,21], phosphorylation potential (PP) [22], and the phosphorus spectral modulus (PSM) [23], all measures of metabolic health.

The intralenticular and intraretinal concentrations of NTP were not only similarly high [24] but also orders-of-magnitude higher than those required to meet the energy demands needed by either lenticular or retinal tissues. The purpose of this report was (1) to compare the high concentration of NTP as a feature in tissues with low and high metabolic activity, (2) to determine the influence of NTP on ratios representing metabolic health in an effort to uncover clues to further elucidate the reason(s) for this amount of NTP, and (3) to integrate biochemical theory, metabolic data, and physiological relevance.

## 2. Experimental Procedures

### 2.1. Tissue Procurement

Pigmented Long Evans rats of both sexes weighing 300 gms were housed in accordance with the guidelines of the Committee on Use of Animals in Research and the Committee on Care and Use of Laboratory Animals of the Chicago College of Osteopathic Medicine, the Committee on Animals of Harvard Medical School, the ARVO Resolution on the Use of Animals in Research, and the guidelines prepared by the Committee on Care and Use of Laboratory Animals of the Institute of Laboratory Resources, National Research Council [DHEW (DHHS) publication No. (NIH) 78-73], which included appropriate and approved periods of quarantine to ascertain the health of the animals. Lenses (n = 24) were pooled in 4 sample groups, each with 6 lenses; retinas (n = 90) were pooled in 3 sample groups, each with 30 retinas. To procure lens tissues, rats were sacrificed using a lethal dose of sodium pentobarbital, their eyes were enucleated and the crystalline lens was extracted *en toto*. To procure neurosensory retinal tissues, rats were sacrificed by decapitation between 0800 and 0900 h to control for possible diurnal variation in metabolites and dissected in oxygenated bicarbonate buffer to minimize altered metabolic status. Lens and retinal tissues were immediately immersed in liquid nitrogen.

### 2.2. Perchloric Acid Extracts

Frozen tissues were collected in NMR sample tubes for lens and retina tissue groups. Experiments were repeated thrice for each group. The frozen tissues were pulverized to a fine powder with a stainless steel mortar and pestle chilled with liquid nitrogen. This preparation was added to a centrifuge tube containing 60% perchloric acid, pre-frozen in liquid nitrogen. Tissue extracts were prepared as described previously [3]. The powder was stirred continuously while it was warmed to a paste consistency and was centrifuged at 43,500× *g* for 15 min at −5 °C. After centrifugation, the supernatant was transferred to an equivalent volume of 10 N KOH, and pH was adjusted to 10. The sample was centrifuged to remove precipitated KCLO_4_. The supernatant was then scrubbed by passing through a potassium Chelexz-100 column to remove alkaline earth and transition metal cations. The column effluent was lyophilized and redissolved in 1.0 mL of 2.0% D_2_O for NMR analysis [3,25,26,27].

### 2.3. Nuclear Magnetic Resonance (NMR) Spectroscopy

A General Electric 500 MHz NMR spectrometer system equipped with deuterium stabilization, variable temperature, and Fourier transform capabilities was operated at 202.4 MHz for ^31^P NMR. A wide-bore Oxford superconducting magnet (11.5 T) was interfaced to the NMR spectrometer system. The extracts were analyzed with and without proton decoupling after placing them in NMR microcell assemblies. Each analysis was conducted extending >24 h to maximize signal resolution.

Although chemical-shift data are reported relative to the usual standard of 85% inorganic orthophosphate (Pi) [27], the primary internal standard was the natural glycerol 3-phosphorylcholine (GPC) resonance of the lens and the retina. GPC has been reported to exhibit a relatively constant chemical shift for phosphate in aqueous solutions (−0.13 δ). This is because GPC is not influenced by variations in pH, ionic strength, or countercation conditions [28,29]. (Note that phosphate chemical-shift values may vary slightly among samples due to preparative procedures used in other laboratories, solutes and counter cations in the sample, and, particularly, pH).

#### Spectrometer Conditions

The spectrometer conditions used in the microcell analysis were as follows: pulse sequence, 1 pulse; pulse width, 18 µs (45º spin-flip angle); acquisition delay, 500 µs; cycling delay, 500 µs; number of scans, 40,900; number of data points per free induction decay, 32,768; acquisition time, 1.36 s; and sweep width, ±6024 Hz. A computer-generated filter time-constant introducing a 0.6 Hz line broadening was applied to each sample.

### 2.4. ^31^P NMR Signals

Data reduction, including peak-area and chemical-shift measurements, was made following standard techniques as previously described [27]. In the case of the rat lens tissue, metabolite values were provided in mole percent phosphate, with a standard error of the mean (SEM) [6]. In the case of the rat retina, tissue metabolite values were provided in nmol/mg protein with SEM as presented in the original data [2]. As such, without a case-by-case measure of each tissue, a statistical comparison is not possible.

### 2.5. Measurement of Metabolic Health Status

Ratios of metabolic health involving nucleoside triphosphate concentrations were computed. Ratios included the energy charge of the adenylate system AEC = ([NTP] + 0.5[NDP]/[NTP + NDP] + [NMP]) [20,21], the phosphorylation potential PP = ([NTP]/[NDP][Pi]) [22], and the phosphorus spectral modulus PSM = (high-energy phosphates)/(low-energy phosphates) [23]. Hereafter, the AEC is designated nucleoside triphosphate energy charge (NEC).

## 3. Results

### 3.1. NMR Spectral Signals and Signal Bands

**Figure 1:**
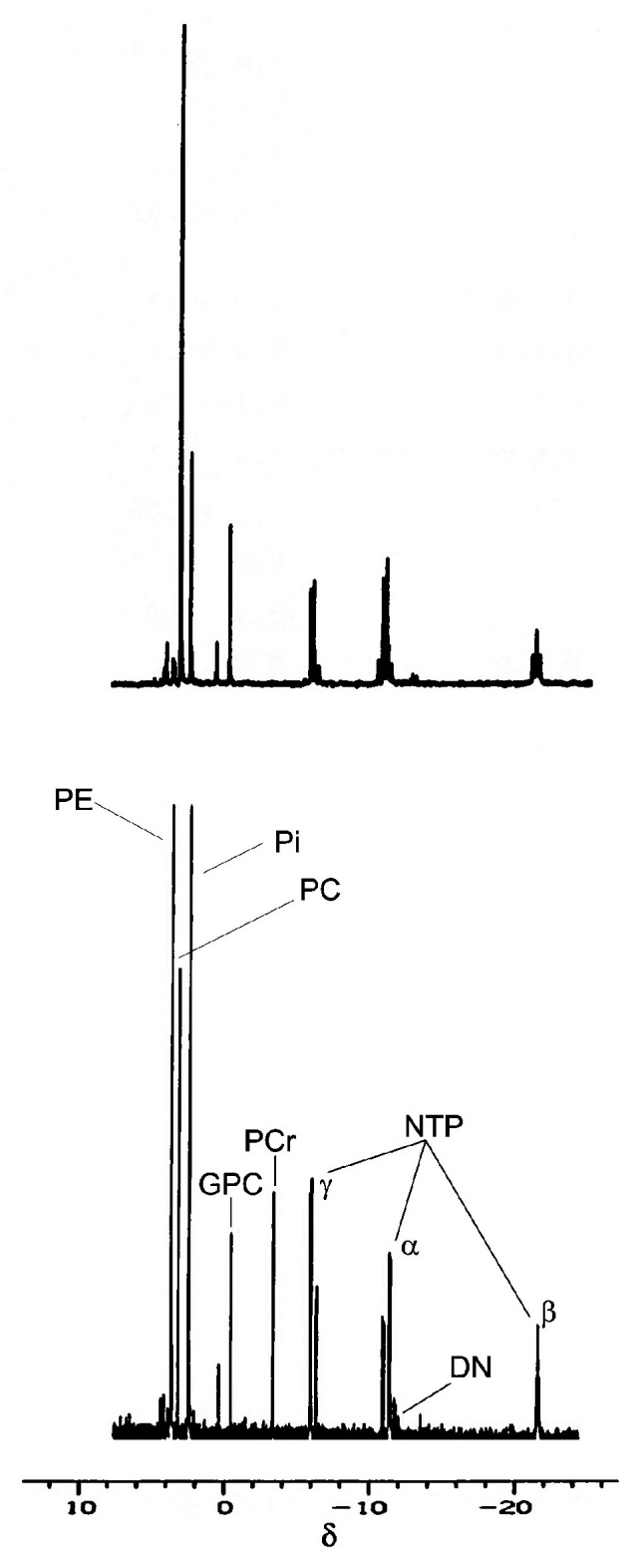
^31^P NMR spectra of perchloric acid extracts of rat lens (top) and rat retina (bottom). The chemical shift δ scale is relative to the resonance position of 85% inorganic orthophosphoric acid (Pi) and is the scale for both the lenticular (top) and retinal (bottom) spectra. Phosphate resonance signals or phosphate resonance signal groups or bands as they occur in their NMR spectral position from left to right: the sugar phosphates: orthophosphate monoesters (4.4 to 3.0 δ) and glycerol 3-phosphate (4.30 δ); phosphorylethanolamine (PE) (3.84 δ) undetected in the lens; phosphorylcholine (PC) (3.33 δ) highest in the lens; adenosine monophosphate (3.73 δ) observed in the retina; inorganic orthophosphate (Pi) (2.60 δ) highest in the lens; glycerol 3-phosphorylethalamine (0.80 δ); glycerol 3-phosphorylcholine (GPC) (−0.13 δ); phosphocreatine (PCr) (−3.10 δ) high in the retina; total nucleoside triphosphates (ATP) [γ (−5.0 δ), α (−10.5 δ), β (−21.5 δ) group phosphates], total nucleoside diphosphates [β (−6.1 δ), α (−10.6 δ) group phosphates]; dinucleotides (DN) (−11.4 to −12.9 δ).

Individual organophosphate NMR spectral signals [glycerol 3-phosphate (Gly 3-P), phosphoethanolamine (PE), phosphocholine (PC), adenosine monophosphate (AMP), inorganic orthophosphate (Pi), glycerol 3-phosphorylethanoamine (GPE), glycerol 3-phosphorylcholine (GPC), phosphocreatine (PCr)] and spectral signal bands grouping classes of organophosphates (orthophosphate monoesters including the sugar phosphates), the nucleoside triphosphates [(NTP) = (ATP + GTP + CTP + UTP)], the nucleoside diphosphates [(NDP) = (ADP + GDP + CDP + UDP)], and the dinucleotides including the nucleoside diphosphosugars (n = 12) were measured as mole percent phosphate (Table 1). These NMR signals representing individual organophosphates and spectral bands are presented in the order in which they appear in the ^31^P NMR spectrum on the δ scale, arranged from low-field to high-field (positive to negative chemical-shift values) (Figure 1), as is recommended by IUPAC [30].

Although the mole percent phosphate data available for both the lens and retinal tissues and the values for the standard error of the mean (SEM) are available, the mole percent phosphate in the case of the retina had to be calculated from nmol/gm protein. This is because the values given for the SEM for the retina were presented from nmole/gm protein data [2]. This being the case, the SEM for the mole percent phosphate values could not be determined for the retina without information for each individual sample run. This information was not presented [2]. As such, it was not possible to perform statistical analyses between the lens and retina. However, when examining the SEM values present for nmole/gm protein (which demonstrate the degree of difference between the sample groups), it is obvious that this difference is quite small. As such, the comparison of mole percent phosphate between lens and retina may be considered acceptable (Table 1). In this case, interpretations of the differences between the mole percent phosphate in the lens and retina should be considered.

### 3.2. Nucleoside Triphosphates

The total NTP was used in this study to represent all the nucleoside triphosphates with the exception that, in the case of the retina, the GTP, CTP, and UTP signals were strong enough to permit measurement [2], whereas in the lens, these other nucleoside triphosphates (GTP, CTP, and UTP) could not be detected using our current NMR instrument magnet strength. Separation of the nucleoside triphosphates (ATP, GTP, CTP, and UTP) in the rat lens was not possible in part due to the ATP signals overlapping the GTP, CTP and UTP resonance signals; thus, they had to be measured together. Likewise, detection of the nucleoside diphosphates (ADP, GDP, CDP, and UDP) was also not possible, and these were likewise measured together, as were the nucleoside monophosphates (AMP, GMP, CMP, and UMP). We assume that the concentration of NTP in the lens and retina could be expected to require a concentration of NTP significantly above basic metabolic needs [3,4]. The NTP concentration in the lens was 41.0% total phosphate [3], whereas, from calculations made in the present paper, it was 37. 6% in the sensory retina.

### 3.3. Other Organophosphates Measured in the ^31^P NMR Spectrum

These metabolomic results are presented in the order that they appear in the ^31^P NMR spectrum. The most notable differences in the ^31^P NMR spectra between lens and retina revealed that among the low-energy phosphates, lens phosphorylcholine (phosphocholine, PhosC) was unexpectedly very high in contrast to the retina, phosphorylethanolamine (PE) was undetectable in the lens, and Pi was more than double the concentration in the retina versus the lens (Table 1). Among the high-energy phosphates, PCr was 15 times higher in the retina than in the lens, NTP concentrations were nearly the same in the lens and the retina, NDP in the retina was more than 2.5 times that found in the lens, and the mole percent of the orthophosphate monoesters and the dinucleotides and nucleoside diphosphosugars was nearly 40% higher in the lens compared to the retina (Table 1).

### 3.4. Computation of Indices of Metabolic Health

Metabolic ratios and indices, where ATP is a principal component, were calculated, including the phosphorus spectral modulus (PSM). Calculation of the metabolic ratios, which are measures of metabolic health, revealed that the lens NTP/NDP was nearly three times that of the retina, the lens NTP/Pi was more than double that of the retina, and retinal PCr/NTP was 16 times greater than that of the lens (Table 2). The metabolic indices involving multiple organophosphates revealed that the NEC (also known as AEC) was similar in both lenticular and retinal tissues, the phosphorylation potential (PP) was nearly seven times greater in the lens, and the ^31^P spectral modulus (PSM) was nearly the same in both the lens and retina (Table 2). The PSM values were 1.28 for the lens and 1.42 for the retina.

## 4. Discussion

This report on the comparative metabolomics of lens and retinal ocular tissues focuses on (1) the high and similar NTP concentrations between these two metabolically diverse tissues, (2) the similar PSM values, which are measures of overall metabolic health status, and (3) further support for the previously unrecognized function of NTP as a hydrotrope in the maintenance of protein solubility and prevention of protein aggregation. Given the above similarities and functions and the fact that this report also extolls the similarities and differences in individual spectral signals and resonance bands of metabolites measured by ^31^P NMR spectroscopy and their activity, the discussion is organized into subsections.

Subsection 1 addresses the comparative analyses of the metabolically quiescent lens and the highly metabolically active retina as they relate to similarly high NTP levels and similar PSM values among these metabolically diverse tissues. Among both lenticular and retinal tissues, NTP is the most highly concentrated of the phosphatic metabolites, and the PSM calculation is inclusive of the entire spectrum of high-energy phosphates and low-energy phosphates in the ^31^P NMR spectrum. Subsection 2 addresses energy production between the structurally and functionally diverse lens and retina. Given this diversity among these lenticular and retinal tissues, Subsection 3 addresses the indices of phosphatic metabolism calculated in this study to include the energy charge of the adenylate system (NEC), the phosphorylation potential (PP), and the phosphorus spectral modulus (PSM). Subsection 4 provides a computational interpretation of the individual metabolites and the resonance bands of metabolites as they relate to lens and retinal tissues. The final subsection, Subsection 5, describes a hypothesis regarding the high NTP levels in both the lens and retina.

### 4.1. Subsection 1. Analyses of Similarly High NTP Concentrations and PSM Values

We present the total concentrations of nucleoside triphosphates in two metabolically diverse ocular tissues, the lens and the retina, which have unexplainably similar NTP concentrations. Among all the organophosphates present in the ^31^P NMR spectrum of the lens [3] and retina [2], NTP has the highest concentration. As mentioned above, the lens is one of the most metabolically quiescent tissues in the body, and the retina is one of the most metabolically active tissues in the body. This phenomenon of similar NTP concentrations in these metabolically divergent tissues is difficult to explain and is peculiar, since the lens exists at the low end of the spectrum of metabolic activity, whereas the retina exists at the high end of the metabolic spectrum. Metabolic similarity with regard to NTP among very different tissues is, however, consistent with our previous observations among metabolically diverse tissues [24]. This is consistent with our previous findings of similarities in the NTP concentrations identified among a range of unicellular and multicellular organisms across the phylogenetic spectrum [24].

#### Diverse Intracellular Functions of NTP

The repertoire of NTP cellular functions is diverse, as described in the Introduction above. The finding of the involvement of NTP and its effects on protein aggregation [11,12] profoundly competes in importance with its classically known function as the principal source of energy powering cellular metabolism. We describe elsewhere the potential for the prevention of protein aggregation and maintenance of protein function [4]. The importance of preventing protein aggregation and maintaining protein function for intracellular metabolic health is critical for cellular homeostasis and life.

##### NTP as a Hydrotrope

This phenomenon of a unique and multifunctional NTP molecule [28] with its enigmatic high concentration, as originally reported in the metabolically quiescent lens [3], defied interpretation until Patel et al. [9] reported on a chemical property of NTP that manifested only at mM concentrations. This postulation was based on the NTP being amphiphilic. As such, NTP is characterized by a hydrophilic tripolyphosphate moiety and a relatively hydrophobic adenine ring. Due to this amphiphilic property, NTP can function as a hydrotrope [10]. NTP can bind to the hydrophobic surface of proteins and potentially maintain intracellular proteins in a fluid state sufficient to prevent protein aggregation [9]. In the fluid (non-aggregated) state, the proteins maintain their programmed biological activity. Conversely, with loss of high millimolar (mM) NTP concentration levels, destructive protein aggregation occurs, with concomitant loss of viable protein function. Patel et al. [9] demonstrated that NTP was much more efficient than classical hydrotropes, creating a microenvironment for hydrophobic molecules. This phenomenon led to the postulation that high concentrations of NTP prevented protein aggregation. This NTP function has been reported in cellular/tissue homogenates [9], and we hypothesize that an elevated NTP concentration inhibits protein aggregation within the living intact lens organ [4]. In the lens, protein aggregation has been shown to be the etiology of cataractogenesis [11,12,13]. Additionally, a similar phenomenon may be at play in the retina and function in preventing the protein aggregation observed in retinal degenerative diseases, such as age-related macular degeneration (ARMD) [11,15,16,17,18].

### 4.2. Subsection 2. Energy Production Among Structurally and Functionally Diverse Tissues

With the similarity in the millimolar (mM) concentration of NTP in the lens and the retina, what are the differences in the metabolic activity in the lens and the retina? The differences in metabolic activity between the lens and the retina are exemplified by first focusing on their energy production. Lenticular metabolism for the production of energy is principally driven by anaerobic glycolysis. Anaerobic glycolysis is necessary because the lens is avascular and appears to rely on the aqueous humor for nutritional support, and with maturation, its cells undergo denucleation with a consequent loss of intracellular organelles, e.g., mitochondria. However, the lens epithelial cells and superficial lens fiber cells of the cortex are nucleated and contain mitochondria and, thus, have some level of aerobic metabolic activity. This is in contrast to the anatomy of the mature lens, where the majority of lens fiber cells of the deeper lens cortex and all of the fiber cells of the lens nucleus are less metabolically active and without mitochondria [31]. Though quiescent metabolically, the tissues of the lens organ having higher metabolic activity compared to the nuclear region of the lens are those of the capsule + epithelium (10.30 mole fraction phosphorus per 100 g tissue), followed by the cortex (0.87 mole fraction phosphorus per 100 g tissue) [32].

The maturing lens fiber cells of the deeper cortex and the entire nucleus are eventually characterized by a homogenous electron-dense appearance by transmission electron microscopy [33], with no visible membrane-bound organelles to produce NTP.

In contrast to the avascular lens, the highly vascularized retina with its dual blood supply is principally driven by aerobic metabolism. This is because all cells of the retina contain mitochondria and thus are principally aerobic. The sensory retina is a relatively densely packed multilayered tissue comprising nucleated neuronal cells with intervening nucleated neuronal networks of fiber connections, the plexiform layers. The most mitochondrial-laden retinal cells are the photoreceptors, where mitochondria are densely localized to the ellipsoid of the inner segments. As such, the photoreceptors are thought to produce the most NTP of the retinal cells; however, a specific quantification of NTP production has not been explicitly determined.

At the metabolic level, a major difference between the lens cells and the neurosensory retina is the more abundant mitochondria, making the retinal cells mitochondrial-rich compared to the mitochondrial-poor lenticular cells. As noted above regarding lens maturation, there is a decline in the population of nucleated cells with an accompanying and coincident decline in the number of mitochondria. This contributes to the mitochondrial-poor status of the lens with its paucity of mitochondria [33]; it is obvious that the lens tissue does not play a natural, highly metabolic role. Conversely, all the neuronal cells of the neurosensory retina develop with mitochondria and intracellular organelles typical of rat retinal cells [34,35]. Although the percentage of lens cells with mitochondria (epithelium and superficial cortex) would be small relative to the deeper cortex and entire nucleus, no quantitative information can be found relative to the estimated 1500 total lens fiber cells in the rat [36].

#### Nucleoside Triphosphates

Since the cytoarchitectural and histological differences between the lens and retina are prodigious, these tissues would be expected to have very different concentrations of NTP. In fact, considering the high metabolic function(s) of the retina, it would be expected that the NTP concentration would be greater than that in the metabolically quiescent lens. One consideration is our report on the presence of a high concentration of guanosine triphosphate (GTP), a triphosphate in addition to ATP that is present in sufficient concentration to serve as another high-energy source in the retina [2]. The presence of the tri-, di-, and monophosphates of guanosine, cytosine, and uridine as distinct spectral signals, as we have reported in the retina [2], is not detected in the lens with current NMR magnet strengths; these spectral signals remain undetectable as they are overlapped by the ATP spectral signals (Figure 1). Metabolically, in terms of high-energy phosphates in the retina, ATP (11.78 nmol/mg protein) and GTP (8.12 nmol/mg protein), though not equivalent, are reasonably precise [2].

### 4.3. Subsection 3. Indices of Phosphatic Metabolism

The ratios of phosphatic metabolism presented herein, as measures of metabolic health, stress, and disease [23], can be divided into single-variable or multivariable indices. Single-variable indices consist of a ratio composed of two spectral signals, e.g., NTP/NDP, NTP/Pi, and PCr/NTP (Table 2). The multivariable indices consist of a ratio involving the interplay of more than two spectral signals as measured by the energy charge of the adenylate system (AEC, also known as NEC), the phosphorylation potential (PP), and the phosphorus spectral modulus (PSM).

#### 4.3.1. Energy Charge of the Adenylate System (AEC)

The AEC, a multivariable index, is indicative of the need for energy metabolism. Even though there may be differences in the rate at which energy is consumed in the highly metabolic retina as opposed to the metabolically quiescent lens, an equilibrium in both the lens and the retinal metabolic energy is maintained in both tissues as the result of this computation, and they are not appreciably different (Table 2). The retina needs more energy to fulfill its metabolic function; having a higher metabolic rate, it is known to consume a significant amount of oxygen, about 20% of the total oxygen used by the brain [37]. Moreover, the retinal photoreceptor cells require high energy for phototransduction, converting light into electrical signals. The overall equilibrium is maintained regarding the energy flux, however, which is about the same.

#### 4.3.2. Phosphorylation Potential (PP)

The phosphorylation potential (PP), another multivariable index, has to do with the intermediates of the oxidative phosphorylation pathway accumulating in the lens and probably not being used appreciably in the retina. The PP has to do with the rate of energy production in the lens and the retina. It remains puzzling as to why the PP is many times higher in the metabolically quiescent lenticular tissue in contrast to the metabolically highly active retinal tissue (Table 2).

#### 4.3.3. Phosphorus Spectral Modulus (PSM)

The more complex multivariable index is the phosphorus spectral modulus (PSM), which we have shown to be a measure of overall cellular, tissue, and organ health [23]. With a PSM > 1, both the lens and retinal tissues examined herein are considered healthy. The nucleoside polyphosphates of high-energy metabolism (ATP, GTP, ADP, GDP) are the major contributing factors to the value of the PSM. This spectral modulus is a more comprehensive indicator of the energy status of the lens than any of the other metabolic indices presented herein (Table 2). This is because the PSM includes the entire spectrum of phosphatic metabolites in its calculation [23].

Of the computed metabolic indices from the 12 individual metabolites or resonance bands of metabolites detected in the lens and retina, only the NEC (also known as AEC) and the PSM were similar (Table 2). The PSM is a ratio that reflects differences in the entire ^31^P spectrum of metabolic data, of which there appears to be minimal or no difference between the lens and the retina. Since the phosphorus spectral modulus (PSM) is an index calculated as the ratio between the entire high-energy phosphate spectral band and the entire low-energy phosphate spectral band [8], the metabolic health status of a tissue can be measured using this index [23]. The PSM provides a gross spectroscopic index of the energy balance in cells, tissues and organs [8]. This index provides a unit for the statistical analysis of interrelations between single phosphorus metabolite signals and metabolite groups (Table 1).

### 4.4. Subsection 4. Interpreting Metabolite Spectral Signals and Signal Bands

In addition to the comparisons of lenticular and retinal nucleoside triphosphates and metabolic indices above, comparisons of the other low-energy phosphates and high-energy phosphates were made. Henceforth, we discuss the individual metabolite spectral signals and signal groups as they are organized in the ^31^P NMR spectra of lens and retina from the low to high magnetic field (Figure 1).

#### 4.4.1. Orthophosphate Monoester Band

Considering the low-energy phosphates, the orthophosphate monoesters (4.4 to 3.0 δ) are indicative of the level of sugar phosphates involved early in intermediary metabolism. Although statistical analysis was not possible without the original retinal data examined case-by-case, the orthophosphate monoester signal band was equivalent in these metabolically divergent tissues (Table 1).

#### 4.4.2. Phosphodiester Precursors

The precursors to the phosphodiesters (3.33 δ), phosphoethanolamine and phosphocholine, are known to play important roles in components of cell membranes [38]. It is surprising that phosphoethanolamine and phosphocholine exhibited a remarkable difference in the lens versus the retina, with phosphoethanolamine being undetected in the lens at our current NMR magnet strength.

Of note, in our studies comparing the concentration of intralenticular phosphocholine among different species, we have demonstrated that phosphocholine is very high in the rat model compared to other animal models [39]. Phosphocholine, with the exception of the NTP, is the most abundant organophosphate in the rat lens [39,40]. This phenomenally high concentration of phosphocholine has also been reported by others [41,42]. Phosphocholine and phosphoethanolamine are among the organophosphates observed to decrease in concentration in cataracts and in lenses subjected to cataractogenic stressors [43,44]; however, this was not observed with other known metabolic stressors [45].

#### 4.4.3. Inorganic Orthophosphate

Inorganic orthophosphate (Pi) (2.60 δ) is much richer in the retina, being more than twice the concentration of that in the lens (Table 1). This is presumed to be because oxidative phosphorylation is much more prominent in the retina than in the lens. Oxidative phosphorylation uses Pi to produce ATP. Oxidative phosphorylation is lower in the lens because less Pi is used. Activity of the high-energy phosphate pathway is greater in the retina than it is in the lens (Table 1). Pi in the retina is elevated, indicating high-energy phosphate is being rapidly consumed. The concentration of Pi increases with tissue decomposition, for example, in aging [19] or in metabolic stress [3,43,44].

#### 4.4.4. Phosphodiesters

The next downfield signals in the NMR spectrum are the phosphodiesters glycerolphosphorylethanolamine (GPE) (0.80 δ) and glycerolphosphorylcholine (GPC) (−0.13 δ) (also designated in the literature as glycerylphosphorylethanolamine and glycerylphosphorylcholine, respectively). These metabolites are key residues in membrane phospholipids and form the backbone of the phospholipid polar head groups of lenticular and retinal membranes.

#### 4.4.5. Phosphocreatine

PCr, another high-energy source, is 15 times higher in the retina than that found in the lens. This observation is consistent, since the retina has a much greater active energy metabolism. This high concentration of PCr is expected since the retina is neural tissue and its concentration of phosphocreatine resembles the high concentration of PCr in the brain [46,47]. In general, metabolically, PCr actually serves as an energy buffer when ATP is being rapidly consumed in the retina. As such, when the ATP concentration starts to decline, the PCr can be employed as a high-energy pool source. The PCr is at a low concentration in the lens (Table 1) most likely because the lens is metabolically inactive.

#### 4.4.6. Nucleoside Diphosphates

The NDP concentration is more than twice as high in the retina compared to the lens (Table 1). This is appropriate, since the retina’s higher metabolic activity is believed to use ATP and GTP in higher concentrations. As a result, NTP may be hydrolyzed at the ribose ester group, resulting in increased NDP that may then be additionally phosphorylated to NTP.

#### 4.4.7. Dinucleotides

The elevated levels of dinucleotides in the lens compared to the retina are curious and may be attributable to an increased demand for NTP production. However, this increase in NTP is likely due to an increased need for NTP’s hydrotropic function [4], as the lens has a low and quiescent metabolic activity. The dinucleotides, NAD and NADP, are the principal sources of protons for the electron transport chain that synthesizes ATP.

### 4.5. Subsection 5. A Hypothesis

The NTP concentration necessary for the energy demands of the lens and retinal tissues is ordinarily measured in µM concentrations of ATP. Given that the lens conserves ATP despite being metabolically inactive, its high ATP levels are likely to serve a purpose beyond energy storage. The presence of similarly high concentrations of NTP in these metabolically divergent tissues, as illustrated in the present report, is consistent with the potentially enormous requirement of a high concentration of NTP in order to function as a hydrotrope in the prevention of protein aggregation. This is especially noteworthy considering the lens and its functions are dependent on its high concentration of proteins, the highest in the body. This observation supports our hypothesis of ATP functioning as a hydrotrope in the prevention of protein aggregation, which would result in cataractogenesis [4] and even age-related macular degeneration [11]. Thus, our observation of similar concentrations of NTP in these two metabolically divergent tissues reveals another clue: NTP’s heretofore unrecognized function as a hydrotrope.

### 4.6. Limitations

Although the percent phosphate measurements among the lens and retina may vary, conclusions regarding phosphatic metabolites must be made with caution, considering the potential influence of tissue dissection techniques and the duration of tissue dissection. Considering that the lens derives its nutrition from the aqueous humor, the lens organ is supported metabolically by being bathed anteriorly by the aqueous humor and at least physically supported posteriorly by the vitreous humor. After animal sacrifice, lenses were procured from the enucleated eyes after bisecting the eyes just posterior to the equator of the globes. The lens was removed with a glass lens loop to avoid any metabolic damage induced by contact with a metal instrument and immediately immersed in liquid nitrogen. This is important to reduce the potential for metabolic decline, which is related to NTP, the highest concentration of the lenticular organophosphates [3]. In contrast, the retina derives its nutrition via a dual blood supply, as noted above. Immediately following enucleation and bisection of the globe, the sensory retina was atraumatically dissected from the eye while being bathed in an oxygenated bicarbonate buffer [2] and immediately frozen in liquid nitrogen. Bathing the retina in a buffer limits potential metabolic damage. Only with experience does this technique of tissue procurement permit limited tissue trauma and a prompt dissection time from death to immersion in liquid nitrogen to halt metabolic decline.

The undetected nucleoside triphosphates, GTP, UTP, and CPT, in the rat lens in contrast to the rat retina need to be addressed. Despite our considerable experience with lens and retinal tissue dissection, post-mortem ATP stability is assumed to be equivalent; however, there remains the possibility of metabolic changes. We do not have an explanation for the fact that the nucleoside triphosphates can be measured in the retina [2], but less in the lens [6], other than difficulty with spectral signal overlap where the signals might be covered by the strong ATP signal, or because their spectral signals are too weak to be detected above the spectral noise

Another limitation of this report, as noted above, is that the retina was procured such that the axonal processes are severed at the optic nerve head during dissection. As such, the full extent of the retinal ganglion cell fibers was not included in our measurements.

Additionally, variations in the methods of analytical measurement of ATP concentration can skew determinations compiled from the comparative literature, as we documented previously [8]. Variations among laboratories in ATP determinations cannot be ignored; however, in our studies, the quality and sophistication of the instrumentation used to make such determinations, as well as the training and expertise of the investigators, was controlled, since all analyses were conducted in the same analytical biochemistry laboratory by the same technical staff. As such, the influence of the aforementioned aspects on interpretations of metabolic data was limited.

Although the lenticular and retinal tissue groups studied are heterogeneous, and these tissues are anatomically very different, they both exhibited similarly high mM concentrations of intracellular ATP. This outcome was not anticipated, since these tissues are clearly different in their known metabolic needs. The intracellular content of the deep cortical and nuclear lens cells has a high degree of homogeneity [8]; in contrast, the retinal cells are unusually specialized and heterogeneous. Similarities in the cells comprising these two tissues include the fact that they are all surrounded by a plasma membrane. These plasma membranes have similar internal machinery, comprised primarily of proteins that carry out similar reactions, and the cells of both tissues can be cultured since they are discrete living entities.

Although the rat model is not necessarily an ideal model for metabolic features, as shown in lenticular studies with reported species differences [39], the utilization of the rat animal model offers economic benefits, including the ease of maintenance of animal colonies. The difficulty in this animal model results in limiting its value as a model for the human. This results in potential misinterpretation as to how the findings relate to the human lens and retina.

Differences in sex were not determined in the present study and may influence the phosphocholine levels.

The retina, with its high glycolytic demand, is linked to the extraordinary sensitivity of photoreceptors detecting as little as a single photon of light [48] and the continuous renewal of photoreceptor outer segment disks by disk shedding [49]. This may be explained in part by considering that the tripolyphosphate signal in the retina comprises major components of ATP, GTP, CTP, and UTP [2], whereas the level of GTP or any of the other bases, e.g., CTP and UDP, is, at least at this time, virtually undetectable in the rat lens spectra [7,39]. Our study on phosphatic metabolites in the retina using HPLC and ^31^P NMR allowed for the quantification of the triphosphates ATP and GTP in the rat retina [2]. In our earlier studies of the rat lens [7,39], we were not able to make this distinction due to the problem of overlapping phosphate signals in the NMR spectrum, which limited our analysis.

A study limitation includes the inability to measure exact numerical comparisons between the lens and retina. This is because the measure of the retinal metabolites cannot be determined from the SEM reported as nmoles/gm protein [2] when converting to mole percent phosphate. As such, even though we were unable to determine the p value differences among the metabolites measured, we were able to at least report percentages of differences among the lenticular and retinal tissues. The reporting of these data is important since the SEM [2] demonstrates a minimal difference. Nevertheless, this observation calls for further studies that are not just descriptive comparisons without statistical inference.

Although some literature now exists relevant to ATP, there is a paucity of reports on the ATP metabolite and its relationship with the prevention of protein aggregation, and additional experimentation regarding the importance of ATP on protein aggregation is required [8,11]. Although we do not provide experimental evidence to address the role of NTPs in preventing protein aggregation, scientific data is accumulating in support of ATP as a hydrotrope [4,6,9]. The comparative data between the lens and retina presented along with the calculated indices of health status further underscores the notion and possible role of ATP functioning as a hydrotrope.

### 4.7. Future Studies

The hypothesis that ATP acts as a hydrotrope preventing protein aggregation and maintaining solubilization of protein needs to be directly tested in both the age-related cataractous lenses and age-related retinal degeneration. These studies are required to assay ATP for its hydrotropic activity, which may include measuring lenticular protein aggregation in homogenates with varying ATP concentrations. This may further support the reason for the low metabolic activity of the lens and its hydrotropic activity. Future studies will require direct experimental evidence relevant to protein aggregation or solubility as a function of ATP or related perturbations in lens-relevant preparations with appropriate controls and quantitative analysis. The simplest model might include the use of the lens organ, where even the living intact lens could be examined with ^31^P NMR. More specifically, such studies may include fluorescence and light scattering procedures performed to evaluate if altering NTP concentrations affects lenticular protein aggregation. While the present report mainly compares nucleoside triphosphates in lens and retinal tissues, the observation of an unusual and very high phosphocholine concentration in the rat lens [39] compared to the retina (Table 1) indicates a need for further interspecies investigation.

## 5. Conclusions

This study demonstrates the presence of similar NTP concentrations in lenticular (metabolically quiescent) and retinal (metabolically active) tissues, which reside at opposite ends of the metabolic spectrum. This secondary analysis provides a substantively novel finding. Moreover, the millimolar concentration of NTP in both the lens and retina is at least three orders of magnitude higher than the micromolar level of NTP necessary to conduct its known roles. This high concentration of NTP has the greatest influence on the indices that involve measures of the health status of these tissues. The comparative result of the calculations of these indices provides insights not apparent when observations are restricted to single or few metabolites. The PSM appears to be most influenced by the NTP that includes impact from the entire ^31^P NMR spectrum. This is likely due to the fact that NTP is most highly concentrated in the lens and retinal tissues. The above findings suggest support consistent with the possible role of NTP independent of cellular/tissue metabolism, such as that required by the hydrotropic model.

## Figures and Tables

**Table 1:** P-31 NMR data from lens and retina PCA extracts.

Phosphate or Phosphate Resonance Group	δ ^1^	Mole % Phosphate	
		Lens ^2^	Retina
Orthophosphate Monoesters ^3^	4.8 to 3.78	3.5 ± 0.2	3.39
Glycerol 3-Phosphate (Gly 3-P)	4.30	1.46 ± 0.2	1.29
Phosphoethanolamine (PE)	3.84	0	7
Phosphocholine (PC)	3.33	21.3 ± 0.5	3.77
Adenosine Monophosphate (AMP)	3.73	0.6 ± 0.07	0
Inorganic Orthophosphate (Pi)	2.60	9.3 ± 0.1	21.81
Glycerol 3-Phosphorylethanolamine (GPE)	0.80	1.4 ± 0.1	0.98
Glycerol 3-Phosphrylocholine (GPC)	−0.13	5.4 ± 0.3	3.07
Phosphocreatine (PCr)	−3.10	0.2 ± 0.08	3
Total Nucleoside Triphosphates (NTP)	−5.0 to −21.5	41 ± 0.5	37.61
Total Nucleoside Diphosphates (NDP)	−6.1 to −10.6	3.2 ± 0.6	8.57
Dinucleotides (DN)	−11.4 to −12.9	11.4 ± 0.17	6.91

^1^ Chemical-shift (δ) or the δ range of resonance groups. ^2^ Mole % phosphate ± standard error of the mean (SEM). ^3^ Orthophosphate monoesters minus Gly 3-P, PE, PC, and AMP.

**Table 2:** ^31^P NMR indices and moduli data from lens and retina PCA extracts.

	Tissue	
	Lens	Retina
Phosphate Indices		
NTP/NDP ^a^	12.81	4.42
NTP/Pi ^b^	4.41	1.72
PCr/NTP ^c^	0.005	0.08
Moduli Derived from Phosphate Mole-Fractions		
Nucleoside Triphosphate Energy Charge (NEC) ^d^	0.95	0.86
Phosphorylation Potential (PP) ^e^	1.38	0.2
Phosphorus Spectral Modulus (PSM) ^f^	1.28	1.42

^a^ NTP/NDP, (nucleoside triphosphates)/(nucleoside diphosphates). ^b^ NTP/Pi, (nucleoside triphosphates)/(inorganic orthophosphate). ^c^ PCr/NTP, (phosphocreatine)/(nucleoside triphosphates). ^d^ NEC, ([NTP] + 0.5[NDP])/([NTP] + [NDP] + [NMP]). ^e^ PP, (NTP)/(NDP)(Pi). ^f^ PSM, (high-energy phosphates)/(low-energy phosphates).

## Data Availability

All data needed to evaluate the conclusions are presented in the paper.
